# Specific and Spillover Effects on Vectors Following Infection of Two RNA Viruses in Pepper Plants

**DOI:** 10.3390/insects11090602

**Published:** 2020-09-05

**Authors:** Saurabh Gautam, Habibu Mugerwa, Sivamani Sundaraj, Kiran R. Gadhave, John F. Murphy, Bhabesh Dutta, Rajagopalbabu Srinivasan

**Affiliations:** 1 Department of Entomology, University of Georgia, 1109 Experiment Street, Griffin, GA 30223, USA; sg37721@uga.edu (S.G.); habibu.mugerwa@uga.edu (H.M.); 2 Department of Entomology, University of Georgia, 2360 Rainwater Road, Tifton, GA 31793, USA; sivamanisundaraj@gmail.com; 3 Department of Plant Pathology and Microbiology, University of California, Riverside, CA 92521, USA; kiran.tnau@gmail.com; 4 Department of Entomology & Plant Pathology, Auburn University, 301 Funchess Hall, AL 36849, USA; murphjf@auburn.edu; 5 Department of Plant Pathology, University of Georgia, 3250 Rainwater Road, Tifton, GA 31793, USA; bhabesh@uga.edu

**Keywords:** cucumber mosaic virus, tomato spotted wilt orthotospovirus, *Myzus persicae*, *Frankliniella fusca*, vector–virus interactions

## Abstract

**Simple Summary:**

Mixed infection of plant viruses is pervasive in agricultural systems. Mixed infection-induced host effects on vector/s fitness are often not well characterized. This study examined how infection of cucumber mosaic virus (CMV) and/or tomato spotted wilt orthotospovirus (TSWV) in pepper plants influenced the preference and fitness of their vectors *Myzus persicae* and *Frankliniella fusca*, respectively. Mixed infection resulted in more severe symptoms when compared with single infection. An antagonistic interaction between CMV and TSWV was also observed, wherein CMV titer was suppressed in mixed-infected compared with singly-infected plants. At instances, mixed-infected plants negatively impacted vector/s fitness more evidently than singly-infected plants. In others, the effects of mixed infection on vector preference or fitness did not vary from single infection. Overall, mixed infection in pepper plants did not enhance vector/s fitness and was not conductive to facilitate CMV and/or TSWV transmission and epidemics. The effects of CMV/TSWV infection extended to specific and non-specific vectors. CMV infection enhanced thrips fitness and TSWV infection enhanced aphid fitness. These spillover effects could result in crosstalk between vectors and viruses in the pathosystem, which in turn could lead to mixed infection. The driving forces of these interactions and the outcomes of these interactions remain to be characterized.

**Abstract:**

Mixed infection of plant viruses is ubiquitous in nature and can affect virus–plant–vector interactions differently than single virus infection. While several studies have examined virus–virus interactions involving mixed virus infection, relatively few have examined effects of mixed virus infection on vector preference and fitness, especially when multiple vectors are involved. This study explored how single and mixed viral infection of a non-persistently transmitted cucumber mosaic virus (CMV) and propagative and persistently-transmitted tomato spotted wilt orthotospovirus (TSWV) in pepper, *Capsicum annum* L., influenced the preference and fitness of their vectors, the green peach aphid, *Myzus persicae* (Sulzer), and the tobacco thrips, *Frankliniella fusca* (Hinds), respectively. In general, mixed infected plants exhibited severe symptoms compared with individually infected plants. An antagonistic interaction between the two viruses was observed when CMV titer was reduced following mixed infection with TSWV in comparison with the single infection. TSWV titer did not differ between single and mixed infection. *Myzus persicae* settling preference and median developmental were not significantly different between CMV and/or TSWV-infected and non-infected plants. Moreover, *M. persicae* fecundity did not differ between CMV-infected and non-infected pepper plants. However, *M. persicae* fecundity was substantially greater on TSWV-infected plants than non-infected plants. *Myzus persicae* fecundity on mixed-infected plants was significantly lower than on singly-infected and non-infected plants. *Frankliniella fusca* fecundity was higher on CMV and/or TSWV-infected pepper plants than non-infected pepper plants. Furthermore, *F. fusca*-induced feeding damage was higher on TSWV-infected than on CMV-infected, mixed-infected, or non-infected pepper plants. Overall, our results indicate that the effects of mixed virus infection on vectors were not different from those observed following single virus infection. Virus-induced host phenotype-modulated effects were realized on both specific and non-specific vectors, suggesting crosstalk involving all vectors and viruses in this pathosystem. The driving forces of these interactions need to be further examined. The effects of interactions between two viruses and two vectors towards epidemics of one or both viruses also need to be examined.

## 1. Introduction

Arthropod vectors facilitate transmission of the majority of plant viruses to their host plants [1,2,3]. The frequency and nature of interactions between viruses, vectors, and host plants determine the success of virus transmission [4,5,6]. Plant viruses have been documented to directly influence the insect vector preference and fitness in varying magnitudes [7,8,9,10,11]. Furthermore, plant viruses are known to alter the plant phenotype and physiology, including changes in pigmentation, plant nutritional quality, phytohormones, defense responses, and profiles of plant-volatile organic compounds (VOCs) [12,13,14]. Such virus-induced effects on host plants could in turn modulate insect vector preference and fitness, favoring efficient virus transmission and spread [5,14,15,16,17,18].

Our current understanding of virus-modulated effects on vector behavior and fitness is predominantly based on pathosystems encompassing a virus, a plant host, and a vector species. However, in nature, mixed virus infection both in host plants and in insect vectors are very common [19]. Mixed virus infection could arise owing to preferential orientation of viruliferous vectors to a host plant that is already infected with a different virus species or following inoculation by a vector that ferries two distinct viruses; of course, in both instances, the inoculated plant has to be susceptible to both viruses [19,20,21,22]. The interactions between two distinct viruses in a mixed infection can be synergistic, neutral, or antagonistic [23]. A synergistic interaction is characterized by an increase in titer of one or both viruses following mixed infection in comparison with single infection [24]. In a neutral interaction, the titer of both viruses following mixed infection is not different when compared with single infection [25]. In contrast, an antagonistic interaction is characterized by reduction in the titer of one or both viruses following mixed infection when compared with single virus infection [22,24]. The resultant phenotype following mixed virus infection can sometimes differentially influence the vector preference and/or fitness compared with single virus infection, thereby affecting the transmission of one or both viruses [20,22,26,27,28,29]. Very few studies have investigated mixed virus infection-modulated fitness benefits when two diverse vectors are involved [20].

The pepper virus pathosystem in the southern United States presented a unique opportunity to assess the effects of single and mixed virus infection on two vectors. Pepper plants in this geographic region are often infected by cucumber mosaic virus (CMV) and tomato spotted wilt orthotospovirus (TSWV) [30,31,32]. *Cucumber mosaic virus* is a species in the genus *Cucumovirus* and family *Bromoviridae*, while *Tomato spotted wilt orthotospovirus* is a species in the genus *Orthotospovirus* and family *Tospoviridae*. CMV is transmitted by over 80 aphid species in a non-persistent manner [32], with *Myzus persicae* (Sulzer) and *Aphis gossypii* (Glover) being the predominant vectors in the southeastern United States. Meanwhile, TSWV is transmitted by 10 thrips species in a persistent-propagative manner [33,34]. The tobacco thrips—*Frankliniella fusca* (Hinds), and western flower thrips—*Frankliniella occidentalis* (Pergande), are efficient vectors of TSWV in the region [33].

With exceptions, non-persistent viruses such as CMV attract and arrest their vectors for brief periods without providing long-term fitness benefits, thereby facilitating rapid virus acquisition and inoculation [2,12,35]. In contrast, persistent-propagative viruses such as TSWV are known to attract and arrest their vectors for longer periods and positively influence vector fitness [9,36]. These complex virus-induced effects on the vectors are modulated by alterations in a gamut of host factors, as stated above [9,12,37]. Overall, these tricomponent (host–vector–virus) interactions seem to facilitate virus spread in each category (non-persistent and persistent propagative). However, it is not clear how these component interactions would be altered when a host is infected with two viruses that are on the opposite ends of the transmission spectrum (non-persistent non-propagative versus persistent propagative).

The goal of this study is to, through a series of experiments, assess how virus-induced host-modulated effects on vector preference and/or fitness vary between single and mixed infection in the pepper virus pathosystem. In the process, this study also aims to assess if virus-induced host-modulated effects following single infection are specific to its vector, or if there are spillover effects extended to a non-specific vector feeding on the same host.

## 2. Materials and Methods

### 2.1. Aphids and Thrips Rearing

*Myzus persicae* clone ‘OUR’, initially collected from potato plants more than thirty years ago and maintained on Chinese cabbage, *Brassica pekinensis* (Ruprecht), was obtained from Juan Alvarez at the University of Idaho, Aberdeen Research and Extension Center, Aberdeen, ID in 2010. The clone has since been maintained at the University of Georgia, Tifton Campus, Tifton, GA on Chinese cabbage in the greenhouse at 25 °C, 60% relative humidity (RH), and 16 h L:8 h D photoperiod. The Chinese cabbage plants were maintained in 10 cm (d) × 8 cm (h) pots filled with a commercial potting mixture (LT5 Sunshine mix; Sun Gro Horticulture Industries, Bellevue, WA, USA). The pots were placed in (47.5 l × 47.5 w × 47.5 h cm^3^) insect-proof cages (Megaview Science Co., Taichung, Taiwan).

The adults of tobacco thrips (*F. fusca)* were collected from peanut flowers at the United States Department of Agriculture Belflower Farm, Tifton, GA in 2009. A thrips colony has since been maintained on non-infected peanut leaflets (cultivar Georgia Green) in thrips-proof Plexiglass cages (Munger cages) [38] in a growth chamber (Thermo Scientific, Dubuque, IA, USA) at 25–30 °C and 14 h L:10 h D photoperiod at the University of Georgia, Tifton Campus, Tifton, GA. Peanut leaflets required for maintaining thrips were obtained from two-to-four-week old seed-grown peanut plants (cultivar Georgia Green) in the greenhouse under the same conditions as stated above.

### 2.2. CMV and TSWV Maintenance

Cucumber mosaic virus (CMV) strain *Fny* was obtained from John Murphy, Auburn University, Auburn, AL, and maintained on pepper, *Capsicum annum* L., seedlings through repeated mechanical inoculation. Two-to-three-week old pepper seedlings (cultivar Revolution) were mechanically inoculated with CMV as per the protocol outlined in Mandal et al. and Shrestha et al. [39,40]. Briefly, 1 g of CMV-infected leaf tissue was ground in 10 mL of phosphate buffer along with celite (Acros Organics, Geel, Belgium) and carborundum (320 grit, Fisher Scientific, Fair Lawn, NJ, USA) at 1 g/100 mL phosphate buffer. Plants were initially dusted with carborundum and the inoculum mixture was uniformly applied to all fully expanded leaves using a cheese cloth (American Fiber & Finishing, Inc., Burlington, MA, USA). The inoculated plants were then washed with water and transferred to insect-proof cages and maintained in the greenhouse under the above-stated conditions. Two-to-three weeks post inoculation, the incidence of CMV infection was confirmed by triple antibody sandwich enzyme linked immunosorbent assay (TAS-ELISA) [41]. The polyclonal capture antibody, detection antibody, and enzyme conjugate were all used at 1:200 dilution based on protocols provided by the manufacturer (Agdia, Elkhart, IN, USA). TAS-ELISA was performed in a 96-well microtiter plate (Nunc Maxisorp, Rochester, NY, USA) along with appropriate two positive and negative control samples in each microtiter plate. The various incubation and washing steps were followed as per the guidelines provided by the manufacturer. One-hour post substrate (PNP) addition, the final absorbance values were measured at 405 nm in a spectrophotometer (Model Elx 800, Bio-Tek, Bad Friedrichshall, Germany). The inoculated plants were considered CMV positive using a threshold of average absorbance value of two negative control samples plus four standard deviations [40]. Foliar tissue weighing one hundred milligrams was used from each plant for TAS-ELISA.

TSWV (GA isolate)-infected tobacco, *Nicotiana tabacum* L., leaves collected from tobacco field in Bowen Farm, University of Georgia, Tifton Campus, Tifton, GA served as the initial source of inoculum. TSWV has since been maintained in tobacco plants through repeated mechanical inoculation (cultivar NC 71). Approximately six-to-eight-week-old tobacco plants were mechanically inoculated using the same protocol described above. Inoculated plants were maintained in insect-proof cages in the greenhouse under the above-stated conditions. Three weeks post inoculation, the TSWV infection status was determined using double antibody sandwich enzyme linked immunosorbent assay (DAS-ELISA) [41]. Both primary antibody and the secondary antibody with enzyme conjugate were used at 1:200 dilution based on the protocols provided by the manufacturer (Agdia, Elkhart, IN, USA). DAS-ELISA was performed in a 96-well microtiter plate along with appropriate two positive and negative control samples in each microtiter plate. The various incubation and washing steps were followed as per the guidelines provided by the manufacturer. One-hour post substrate (PNP) addition, the final absorbance values were measured at 405 nm in a spectrophotometer. The inoculated plants were considered TSWV positive using a threshold of average absorbance value of two negative control samples plus four standard deviations [40]. Foliar tissue weighing one hundred milligrams was used from each plant for DAS-ELISA.

### 2.3. CMV and TSWV Titer Estimation in Pepper Plants

Two to three pepper seeds (cultivar Revolution) were planted in 10 cm (d) × 8 cm (h) pots filled with the potting mix. One week later, emerging seedlings were thinned to one/pot. Approximately 10 cm tall three-week-old pepper seedlings with at least three true leaves were mechanically inoculated with CMV and/or TSWV according to established protocols [39,40]. To generate mixed infected pepper plants, non-infected pepper plants were simultaneously mechanically inoculated with CMV and TSWV. The inoculated plants were placed in insect-proof cages and maintained in the greenhouse under the conditions described above. Three weeks post inoculation, the incidence of CMV and TSWV infection was tested by TAS-ELISA and DAS-ELISA, respectively, as stated above. Ten plants representing each treatment viz., CMV-infected, TSWV-infected, and mixed (CMV and TSWV)-infected were used for titer estimation. Ten non-inoculated pepper plants served as negative controls. This experiment was conducted three times (*n* = 30 plants for each treatment).

### 2.4. Myzus Persicae Settling Assay

*Myzus persicae* settling assay was conducted with four treatments viz., non-infected, CMV-infected, TSWV-infected, and mixed (CMV and TSWV)-infected approximately six-week-old pepper plants, following the method described by Castle et al. with few modifications [42]. Briefly, a 10 cm Petri dish lid served as the platform, and four similar sized pepper leaves from four treatments, while remaining attached to their respective plants, were placed diagonally and equidistantly from the center. The leaves were held in place with the help of a Mylar ^®^ film cylinder with four slits for four treatments. A 10 cm tall polyethylene tube with 0.8 cm diameter was inserted into the center of the Petri dish lid that served as the platform. One hundred second or third instar nymphs were transferred to a 0.7 cm diameter glass vial and inserted at the bottom of the polyethylene tube. The aphids were allowed to climb up the polyethylene tube, reach the platform, and settle on pepper leaflets. The set up was left undisturbed in the laboratory at 25 °C for 24 h. After 24 h, aphids settling on each leaf representing each treatment were enumerated. The experiment had six replications, and each replication was conducted with a separate set up. The positioning of leaves representing various treatments was randomly assigned for each replication to avoid any bias. This experiment was repeated once more (*n* = 12 for each treatment).

### 2.5. Myzus Persicae Fitness

*Myzus persicae* developmental time from first instar nymph to adult and fecundity was studied in clip cages. *M. persicae* adults from Chinese cabbage plants were individually clip caged onto non-infected or CMV and/or TSWV-infected plants for 48 h. Then, the adult and all but one nymph from each clip cage were removed with a paint brush. The single remaining nymph was monitored daily and developmental time from nymph to adult was recorded. The nymphs larviposited by the subsequent generation adults were counted and removed daily, and total lifetime fecundity was recorded. Six clip cages (@ one aphid per cage) on six plants of each treatment were included in the experiment. The experiment was repeated once more with six additional clip cages and plants (*n* = 12 clip cages for each treatment).

### 2.6. Frankliniella Fusca Fecundity

Fecundity of adult *F. fusca* on CMV and/or TSWV-infected and non-infected pepper leaves was estimated. Ten adult-female thrips reared on peanut foliage were allowed to oviposit for 72 h on pepper leaves of each treatment placed individually on a Plexiglass cage with moistened filter paper at the bottom. To encourage initial thrips feeding, pine pollen (~0.05 g) was slightly dusted on all treatments by gently tapping a #2 paint brush dipped in a 10 mL glass vial containing pollen twice on each plant. The Plexiglass cages were secured with binder clips to prevent any escape. After 72 h, the adult thrips were removed from the cages. The number of eggs laid on each leaf was counted following acid fuchsin staining based on established protocols [9,43]. Briefly, pepper leaves were placed in a solution containing glacial acetic acid, lactic acid, and ethanol at 60 °C until the leaves were bleached colorless. The leaves were then transferred to lacto-phenol solution containing acid fuchsin high purity stain (Acros Organics, Morris Plains, NJ, USA). After a few minutes, the leaves were washed with water. The leaves were then placed under a dissecting microscope (Meiji Techno, Santa Clara, CA, USA) and stained eggs were counted. Ten leaves were used for each treatment, and the experiment was repeated once more (*n* = 20 for each treatment).

### 2.7. Frankliniella Fusca Feeding

*Frankliniella fusca* feeding damage was estimated at three-day intervals based on the protocol outlined in Sundaraj et al. [44]. Approximately five six-week-old plants of each treatment, non-infected and CMV and/or TSWV-infected, were placed in insect-proof cages (one treatment/cage) in the greenhouse, as mentioned above. In order to encourage initial thrips feeding, pine pollen was dusted on each plant as described above. Ten thrips were released per plant. Thrips feeding injuries were monitored every three days for up to eighteen days. Each plant was carefully examined visually and feeding injuries were recorded. Feeding injuries were used to calculate the feeding damage index for each treatment based on the method outlined by Maris et al. with slight modifications [36]. Thrips feeding injuries were rated based on an arbitrary scale ranging from zero to three, with zero representing no scorable feeding injuries and three representing most injuries (>99% leaf area with thrips feeding scars). Feeding damage indices for each time interval were assessed for each treatment.

### 2.8. Statistical Analyses

Data analyses were performed using R Version 3.4.2 [45]. Before analysis, the data from experiment repeats were pooled. Data for virus titer in infected plants, aphid fecundity and settling, and thrips fecundity and feeding damage were analyzed using linear mixed effect model in ‘Lme4’ package in R [46]. Treatments were considered as fixed effects and replications and experiment repeats were considered as random effects. To meet the assumptions of normality and homoscedasticity of variance, virus titer and fecundity data were log transformed, aphid settling data were subjected to logit transformation, and thrips damage index data were log(x + 1) transformed. Differences in virus titer in infected pepper plants were compared using pairwise contrasts in emmeans package [47]. Aphid settling and fecundity, as well as thrips fecundity, were analyzed using one-way analysis of variance (ANOVA). Thrips damage index data were analyzed using one-way repeated-measures ANOVA. Post-hoc analyses were performed using the ‘emmeans’ package with the default Tukey’s honest significant difference (Tukey HSD) post-hoc test [47]. Treatment means were considered significant at *p* < 0.05. Data pertaining to aphid developmental time were analyzed using a non-parametric Kruskal–Wallis test.

## 3. Results

### 3.1. CMV and TSWV Symptoms and Titer Estimation in Pepper Plants

**Figure 1:**
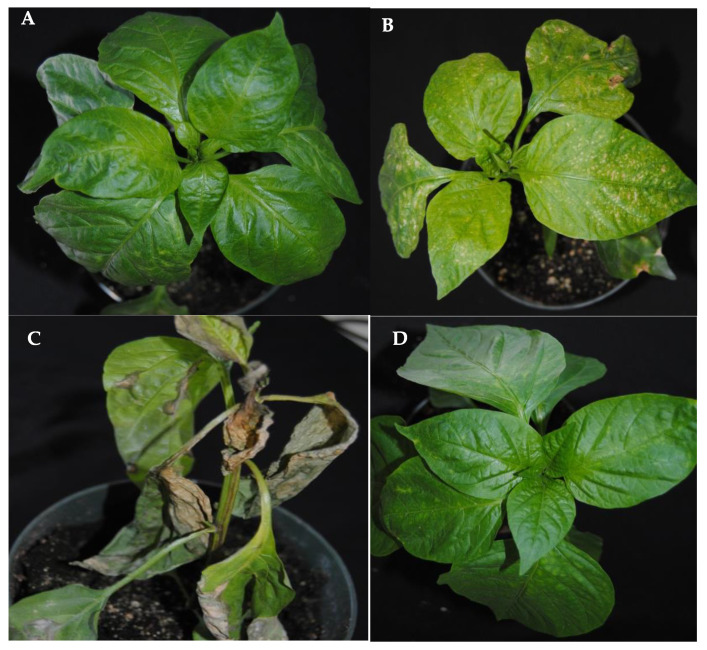
Photographs representative of (**A**) cucumber mosaic virus (CMV)-infected, (**B**) tomato spotted wilt orthotospovirus (TSWV)-infected, (**C**) mixed (CMV and TSWV)-infected, and (**D**) non-infected pepper plants. Virus-infected plants were obtained by mechanically inoculating non-infected plants with either CMV and/or TWV-infected leaf tissue ground in phosphate buffer along with celite and carborundum. Non-infected plants were mechanically inoculated with non-infected leaf tissue ground in phosphate buffer along with celite and carborundum. Photographs were taken approximately four weeks post mechanical inoculation.

**Figure 2:**
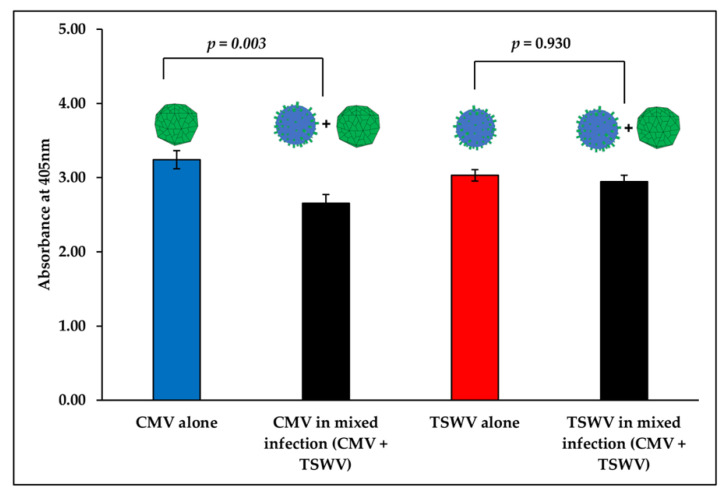
CMV and TSWV accumulation in singly-infected (CMV or TSWV) versus mixed (CMV and TSWV)-infected pepper plants. Bars with standard errors represent average absorbance (optical density, OD) values of samples measured at 405 nm in a spectrophotometer. Virus accumulation in singly-infected (CMV (blue bar) or TSWV (red bar)) versus mixed (CMV and TSWV)-infected (black bar) pepper plants was compared using OD values measured via enzyme linked immunosorbent assay (ELISA). Three weeks post mechanical inoculation, the CMV and TSWV infection status in pepper plants was tested by triple antibody sandwich (TAS)-ELISA and double antibody sandwich (DAS)-ELISA, respectively. Significant differences between treatment means were separated by Tukey’s honest significant difference (HSD) post-hoc test at α = 0.05.

CMV-infected pepper plants displayed mild mottling on younger leaves, and symptoms on TSWV-infected plants included yellowing, chlorotic spots, and ring spots on young and older leaves (Figure 1). Mixed (CMV and TSWV)-infected plants, in addition to CMV and TSWV symptoms stated above, displayed excessive wilting and died prematurely between four to six weeks post mechanical inoculation (Figure 1). CMV titer in mixed (CMV and TSWV)-infected pepper plants was significantly lower than in CMV-infected plants (*F*_1,58_ = 3.56, *p* = 0.003) (Figure 2). In contrast, TSWV titer did not differ between mixed (CMV and TSWV)-infected and TSWV-infected pepper plants (*F*_1,58_ = 0.59, *p* = 0.930) (Figure 2).

### 3.2. Myzus Persicae Settling Assay

**Figure 3:**
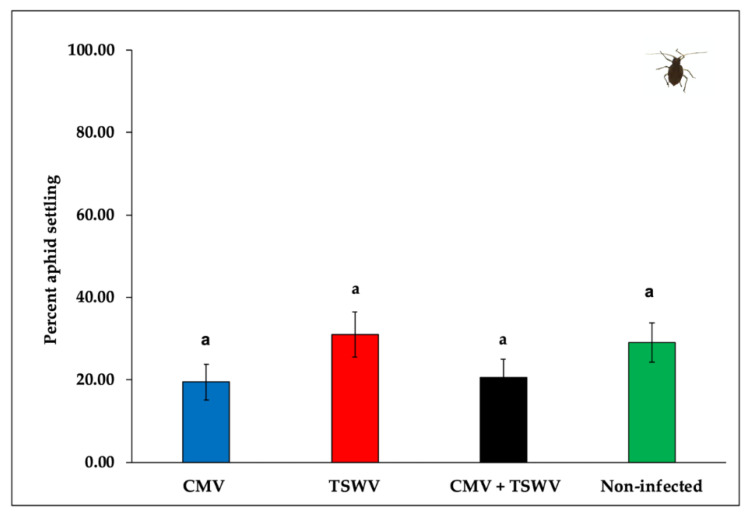
*Myzus persicae* settling on CMV- and/or TSWV-infected and non-infected pepper plants. Bars with standard errors represent average percent settling of *M. persicae* nymphs on CMV- and/or TSWV-infected and non-infected pepper plants 24 h after release. Different letters on bars indicate significant differences between means separated by Tukey’s HSD post-hoc test at α = 0.05. Blue, red, black, and green bars represent settling of aphids on CMV-, TSWV-, mixed-, and non-infected pepper plants, respectively.

*Myzus persicae* did not show any significant settling preference towards CMV- and/or TSWV-infected plants when compared with non-infected plants *(F*_3,44_ = 1.84, *p* = 0.150). Approximately 19.40% of aphids settled on CMV-infected plants, 31.00% of aphids settled on TSWV-infected plants, 20.50% of aphids settled on mixed (CMV and TSWV)-infected plants, and 29.03% of aphids settled on non-infected pepper plants, respectively (Figure 3).

### 3.3. Myzus Persicae Fitness

**Figure 4:**
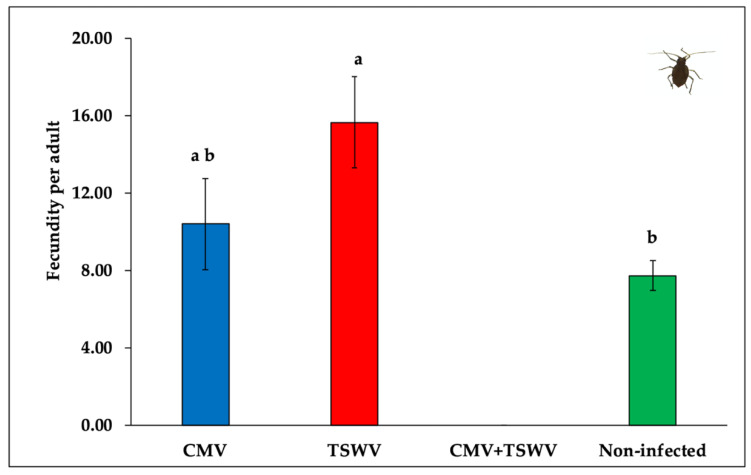
*Myzus persicae* fecundity on CMV- and/or TSWV-infected and non-infected pepper plants. Bars with standard errors represent mean number of nymphs laid by a single aphid in its lifetime on CMV- and/or TSWV-infected and non-infected pepper plants. Different letters on bars indicate significant differences between means separated by Tukey’s HSD post-hoc test at α = 0.05. Blue, red, black, and green bars represent fecundity of aphids on CMV-, TSWV-, mixed-, and non-infected pepper plants, respectively.

The median developmental time from first instar nymph to adult did not differ between aphids developing on CMV- and/or TSWV-infected and non-infected pepper plants (χ^2^_3,44_ = 6.06, *p* = 0.100) (Table 1). However, aphids produced a significantly greater number of nymphs on TSWV-infected pepper plants compared with non-infected pepper plants (*F*_2,33_ = 4.25, *p* = 0.023) (Figure 4). Aphid fecundity did not differ between CMV-infected and non-infected pepper plants. Aphids placed on mixed (CMV and TSWV)-infected plants did not produce nymphs, presumably owing to severe symptoms and premature death. 

### 3.4. Frankliniella Fusca Fecundity

**Figure 5:**
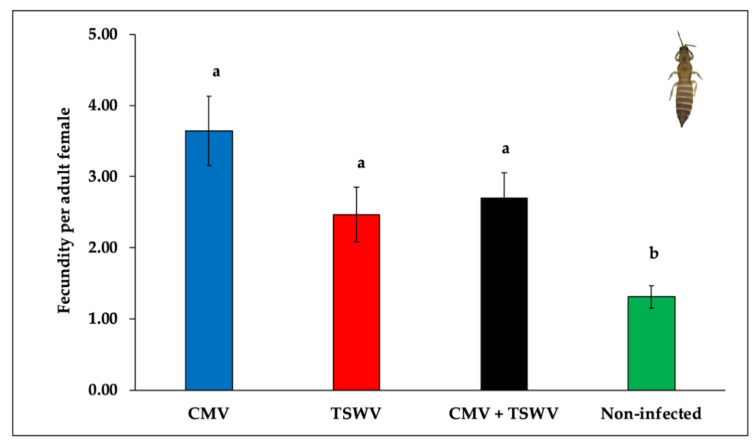
*Frankliniella fusca* fecundity on CMV- and/or TSWV-infected and non-infected pepper plants. Bars with standard errors represent mean number of eggs laid by single adult-female thrips in 72 h on CMV- and/or TSWV-infected and non-infected pepper plants. Different letters on bars indicate significant differences between means separated by Tukey’s HSD post-hoc test at α = 0.05. Blue, red, black, and green bars represent mean number of eggs laid by single female thrips on CMV-, TSWV-, mixed-, and non-infected pepper plants, respectively.

Significant differences were observed in the mean number of eggs laid by female thrips on CMV- and/or TSWV-infected or non-infected pepper plants (*F*_3,76_ = 6.03, *p* = 0.001). Thrips laid a significantly greater number of eggs on CMV- and/or TSWV-infected pepper when compared with non-infected pepper plants (Figure 5).

### 3.5. Frankliniella Fusca Feeding

**Figure 6:**
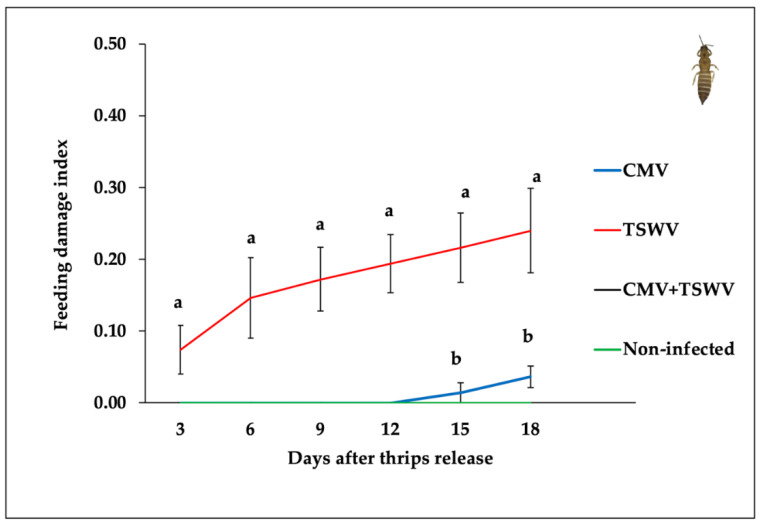
*Frankliniella fusca* feeding damage indices on CMV- and/or TSWV-infected and non-infected pepper plants. Lines with standard errors represent mean feeding damage indices on CMV- and/or TSWV-infected and non-infected pepper plants. Different letters on lines indicate significant differences between means separated by Tukey’s HSD post-hoc test at α = 0.05. Blue, red, black, and green lines represent thrips mean leaf feeding damage indices on CMV-, TSWV-, mixed-, and non-infected pepper plants, respectively.

Scorable feeding damage was only recorded on CMV-infected and TSWV-infected pepper plants, but not on mixed-infected and non-infected pepper plants (Figure 6). The feeding damage indices from CMV- and/or TSWV-infected and non-infected pepper plants varied at 3 days (*F*_3,16_ = 4.82, *p* = 0.014), 6 days (*F*_3,16_ = 6.77, *p* = 0.003), 9 days (*F*_3,16_ = 14.91, *p* < 0.001), 12 days (*F*_3,16_ = 23.14, *p* < 0.001), 15 days (*F*_3,16_ = 17.99, *p* < 0.001), and 18 days (*F*_3,16_ = 14.18, *p* < 0.001) post thrips release. The feeding damage indices at all time intervals were higher on TSWV-infected pepper plants than on CMV-infected, mixed-infected, and non-infected plants (Figure 6).

## 4. Discussions

Mixed virus infection in agricultural systems has been sparsely explored compared with single virus infection in terms of vector preference and fitness, especially when multiple vectors are involved. This study examined if and how single and mixed infection of CMV and TSWV in a common host plant (pepper) influence the preference and fitness of *M. persicae* and *F. fusca*, the predominant vectors of CMV and TSWV in southeastern United States, respectively. Mixed (CMV + TSWV)-infected plants exhibited more severe symptoms than singly-infected plants and most died prematurely. Consequently, at instances, mixed-infected plants were not amenable to study effects on vector fitness. Alternatively, when amenable, the effects of mixed-infected plants on vectors’ preference and/or fitness did not vary drastically from effects of singly-infected plants on vectors. Additionally, this study documented spillover effects of virus infection on a vector of a different virus (non-specific vector) in the same pathosystem. For instance, *M. persicae* fecundity on TSWV-infected plants was higher than on non-infected pepper plants. Moreover, the fecundity of *F. fusca* did not differ between CMV- and TSWV-infected pepper plants, but it was higher when compared with non-infected plants. These spillover effects following single virus infection could in turn facilitate mixed virus infection and ultimately affect the epidemics of one or both viruses.

Virus–virus interactions following mixed infection in host plants range from antagonism to synergism [26,29,48,49]. In the current study, TSWV infection appeared to suppress CMV accumulation in mixed infected pepper plants. These results are in contrast with several other mixed infection studies conducted earlier. For instance, CMV interactions with potyviruses such as potato virus Y (PVY) in tomato and zucchini yellow mosaic virus (ZYMV) in cucumber were synergistic, and CMV accumulation in tissues of mixed-infected plants was higher than in tissues of singly-infected plants [50,51,52]. Increased CMV accumulation following mixed infection with potyviruses was attributed to host defense suppression mediated by the helper component-proteinase (HC-Pro) [53]. Similarly, synergistic interaction between TSWV and a crinivirus—tomato chlorosis virus (ToCV)—in mixed infected tomato plants was observed. Mixed-infected tomato plants had higher ToCV accumulation and unaltered levels of TSWV than singly-infected plants [54]. The results from this study and earlier studies illustrate context-specificity in the nature of virus–virus interactions in mixed infected plants. These interactions could be influenced by numerous host plant and virus factors.

Despite the altered phenotype in virus-infected plants (more so in mixed-infected than singly-infected pepper plants) than non-infected plants, *M. persicae* did not preferentially settle on CMV- and/or TSWV-infected pepper plants over non-infected plants. Earlier studies have reported increased attraction of *M. persicae* and/or *A. gossypii* towards CMV-infected squash and tobacco plants over non-infected plants [12]. The discrepancy between the present and previous studies could possibly be owing to differences in virus strains and host plants and limitations in experimental set up [55,56]. The differences in experimental methods adopted could also influence aphid preference. For instance, the current study assessed settling differences 24 h after aphid release, whereas preference towards CMV-infected over non-infected plants was observed much sooner in an earlier study [12]. Short-term preferential settling towards infected plants and rapid dispersal with no long-term benefits could be conducive for quick acquisition and inoculation of non-persistent viruses such as CMV [12]. The fecundity of aphids did not decrease on CMV-infected plants over non-infected plants in this study. This result is in contrast to outcomes in earlier studies that revealed a substantial fitness reduction of *M. persicae* on CMV-infected plants compared with non-infected plants, and attributed to decreased nutritional quality in infected plants compared with non-infected plants [12,57,58]. Another recent study involving *A. gossypii* and a non-persistent virus, papaya ring spot virus (PRSV), documented increased fitness benefits to aphids on infected plants, possibly facilitated by an enhanced host nutritional profile in virus-infected plants [37]. These results suggest that the effects of non-persistent viruses on their vectors could either be short-term or long-term, and that they could range anywhere in a continuum from reduced fitness benefits to neutral effects to enhanced fitness benefits. Each of these outcomes could facilitate the transmission of non-persistent viruses via different mechanisms (12,35). The mixed-infected plants in this study were highly symptomatic and succumbed to the viruses prematurely. Consequently, they were not conducive to evaluate fecundity. The aphids in this study reached adulthood on mixed infected plants, but they did not produce nymphs, or the newly laid nymphs did not survive owing to poor host quality.

Fitness benefits were also associated with the second vector (thrips) in this study, and they did not vary between singly-infected and mixed-infected plants. The fecundity of *F. fusca* on CMV- and/or TSWV-infected pepper plants was greater in comparison with non-infected plants. Earlier studies have reported that TSWV-infected tomato, pepper, and peanut plants had higher levels of free amino acid in their leaf tissue compared with non-infected plants [9,59,60]. Furthermore, CMV-infected plants also had enhanced free amino acid levels in epidermal and mesophyll cells than non-infected plants [61]. Free amino acids are known to be vital for egg production [9,62,63]. Therefore, it is possible that increased fecundity of thrips on CMV- and/or TSWV-infected pepper plants could be influenced by higher amino acid levels in leaf tissue from infected plants than non-infected plants. Thrips feeding was minimal to non-existent in non-infected pepper plants. The thrips used in this experiment were reared on peanut foliage for multiple generations and were exposed to pepper for the first time. Reciprocal feeding experiments have shown that thrips adults do not perform well when transferred to new host plants [64]. While it might have been ideal to use a *F. fusca* population that is acclimated to pepper plants in this study, repeated attempts to rear such a pepper-acclimated *F fusca* population were not successful. Besides, the current experimental setup is a true representation of natural conditions in parts of southeastern United States. *Frankliniella fusca* foliar feeding on pepper under field conditions is often less severe, despite that *F. fusca* can transmit TSWV to pepper following dispersal from the peanut crop in the farmscape. Taken together, these results suggest that increased nutritional quality of TSWV- or CMV-infected plants might have served as an incentive for *F. fusca* to oviposit and feed on an otherwise unpreferred/unaccustomed host, thereby further facilitating TSWV acquisition and/or inoculation.

In addition to virus-induced host-modulated fitness effects to specific vectors, this study also documented spillover effects to non-specific vectors. As discussed above, thrips performed better on CMV-infected plants over non-infected pepper plants. Similarly, *M. persicae* produced significantly more nymphs on TSWV-infected pepper plants compared with non-infected pepper plants. Perhaps, it is plausible that some virus-induced host phenotype modulations such as the increase in free amino acids and soluble carbohydrates could be somewhat generic and prompt crosstalk between virus-infected host plants and non-specific vectors. In fact, such generic effects following virus infection have been documented to even influence other non-vector herbivores [59,60]. Virus-induced host phenotype-modulated increased attraction and/or fitness benefits extended to non-specific vectors could result in mixed infection. However, it is not clear as to how these mixed-infected hosts will further enhance epidemics of one and/or both viruses. In this study, the resultant phenotype following mixed infection had severe symptoms compared with single infection, resulting in premature death of those plants and limiting the conduciveness to effectively serve as inoculum sources. These results are contradictory to earlier studies with mixed infection, wherein mixed-infected plants were as good as or better than singly-infected plants in influencing their vectors [20,22,28]. These results reiterate context specificity and emphasize that phenotypic alterations in host plants could be the major driving forces in modulating vector responses.

## 5. Conclusions

Interactions between polyphagous vectors and viruses with broad host range frequently lead to mixed virus infection. The resulting mixed virus infection increases the complexity of vector–virus–host interactions in that pathosystem and unraveling such interactions could be tedious. However, as mixed infection is becoming increasingly common in agricultural landscapes, studies on these pathosystems are warranted. The results from this study indicate that virus infection could elicit generic responses that influence non-specific vectors and that crosstalk could lead to mixed infection. The resulting mixed infection could have two outcomes: (1) mixed-infected plants could become dead-end hosts for one and/or both viruses owing to symptom severity (unfit outcome); (2) mixed infected plants could become better inoculum sources and vector/s reservoirs and facilitate virus epidemics better than singly-infected plants (fit outcome). While there is prior evidence supporting the second outcome, this study supports the first outcome. Future studies could concentrate on understanding the intricacies involved, such as what are the driving forces that render a fit outcome versus an unfit outcome? What are the incidence frequencies of fit and unfit outcomes? Are fit outcomes more often associated with one vector and unfit outcomes with multiple vectors?

## Figures and Tables

**Table 1:** Developmental time of aphids on cucumber mosaic virus (CMV)- and/or tomato spotted wilt orthotospovirus (TSWV)-infected and non-infected pepper plants.

Treatments	N ^a^	Nymph-Adult ^b^
CMV	45	8 (6–9) ^a^
TSWV	23	9 (7–10) ^a^
CMV and TSWV	26	8 (7–9) ^a^
Non-infected	30	9 (7–9) ^a^

^a^ Number of nymphs monitored to adulthood. ^b^ Median developmental time (days) from nymph to adult with range in parentheses. Same letters indicate no significant difference (*p* < 0.05). CMV—cucumber mosaic virus. TSWV—tomato spotted wilt orthotospovirus.
